# Population-Based Rates of Revision of Primary Total Hip Arthroplasty: A Systematic Review

**DOI:** 10.1371/journal.pone.0013520

**Published:** 2010-10-20

**Authors:** Kelly L. Corbett, Elena Losina, Akosua A. Nti, Julian J. Z. Prokopetz, Jeffrey N. Katz

**Affiliations:** 1 Department of Orthopedic Surgery, Orthopedic and Arthritis Center for Outcomes Research, Brigham and Women's Hospital, Boston, Massachusetts, United States of America; 2 Division of Rheumatology, Immunology and Allergy, Brigham and Women's Hospital, Harvard Medical School, Boston, Massachusetts, United States of America; 3 Department of Epidemiology, Harvard School of Public Health, Boston, Massachusetts, United States of America; 4 Department of Biostatistics, Boston University School of Public Health, Boston, Massachusetts, United States of America; Hôpital Cochin, France

## Abstract

**Background:**

Most research on failure leading to revision total hip arthroplasty (THA) is reported from single centers. We searched PubMed between January 2000 and August 2010 to identify population- or community-based studies evaluating ten-year revision risks. We report ten-year revision risk using the Kaplan-Meier method, stratifying by age and fixation technique.

**Results:**

Thirteen papers met the inclusion criteria. Cemented prostheses had Kaplan-Meier estimates of revision-free implant survival of ten years ranging from 88% to 95%; uncemented prostheses had Kaplan-Meier estimates from 80% to 85%. Estimates ranged from 72% to 86% in patients less than 60 years old and from 90 to 96% in older patients.

**Conclusion:**

Data reported from national registries suggest revision risks of 5 to 20% ten years following primary THA. Revision risks are lower in older THA recipients. Uncemented implants may have higher ten-year rates of revision, regardless of age.

## Introduction

Total hip arthroplasty (THA) is an efficacious and cost-effective intervention for reducing pain and improving function in patients with advanced hip arthritis [1], [2], [3], [4]. Long-term studies of THA recipients have generally shown that the probability of surviving without undergoing a revision THA exceeds 90% at ten years and 80% at 25 years [1], [5], [6], [7], [8], [9]. However, most of these reports have been from single referral centers or single surgeons, and most refer to only one type of implant [1], [7], [9], [10], [11]. Large national studies, especially population-based studies, provide a better framework for estimating implant survival rates that can more readily be generalized to the majority of patients receiving THA.

While randomized controlled trials are still considered the gold standard for evaluating medical outcomes, trials are both cost-prohibitive and impractical for evaluating the risk of long-term THA revision [2]. National or regional joint replacement registries have the potential to fill this gap in our understanding of long-term THA outcomes. Primary goals of registries include providing data on utilization patterns of total joint replacement and identifying risk factors for poor outcomes and poorly performing devices [2]. The national hip arthroplasty registries from Finland (origination date: 1980), Norway (1987), and Sweden (1979) have been crucial in defining the risks of subsequent revision surgery. By providing feedback to the healthcare community and identifying specific implants with poor results, the registries have also helped to improve the outcomes of THA [12]. Over the past few years, several additional countries have begun national joint registries: Denmark (1994), New Zealand (1997), Hungary (1998), Australia (1998), and Canada (2001).

The national THA registries have produced substantial research on the outcomes and failures of hip arthroplasty from individual countries. However, to our knowledge, the literature on the long-term revision rates following THA in national samples has not been reviewed systematically. Revision data, particularly from national samples, are needed to guide discussions of implant longevity and the risk of revision for elective THA. Health policymakers also need access to such data to anticipate revision volume and associated costs. The goal of this review is to summarize published data on primary THA revision rates over ten years in large national community-based or population-based studies. Additionally, we examine the influence of patient age and prosthesis fixation technique on THA revision rates.

## Methods

*Note: The protocol for this trial and supporting CONSORT checklist are available as supporting information; see PRISMA Checklist S1 and PRISMA Flow Diagram S1.*

### Search Strategy

We conducted a PubMed search to identify studies written in English that were published between January 2000 and August 2010. We did not include articles published prior to 2000 in order to reduce heterogeneity in biomaterials and process of care. We restricted the review to articles published in the peer-reviewed literature to ensure a high level of rigor and quality. We used PubMed hip arthroplasty MeSH Term keywords in combination with search terms relating to revision rates and prosthesis survival and failure. Our verbatim search query, performed August 10, 2010, was:

*(“Arthroplasty, Replacement, Hip/methods”[MAJR] OR “Arthroplasty, Replacement, Hip/statistics and numerical data”[MAJR]) AND (“revision rates” OR “revision rate” OR “rates of revision” OR “rate of revision” OR “prosthesis failure” OR “prosthesis survival”)*

We screened the title and abstract of each article identified for relevance to this literature review. To be included for further review, the studies had to discuss primary hip prosthesis failure leading to revision, provide long-term follow-up, and represent either population- or community-based samples. Single surgeon series, single hospital series, and collaborations between referral centers were excluded based on review of abstracts. Studies reporting only on specific failure mechanisms (e.g. dislocation or infection) were excluded, as were studies focused exclusively on either stem or cup failure (as opposed to failure of *any* component). For abstracts that passed this screening, the full length articles were retrieved and reviewed.

To ensure comparability of articles, minimize bias due to truncated follow-up, and incorporate the methods used in the majority of papers reporting prosthesis survival, we chose ten-year revision-free survival estimated by the Kaplan-Meier method as the primary outcome for our review. A key advantage of the Kaplan-Meier method is that it accounts for all persons who were lost to follow-up or died. We excluded studies that did not report the Kaplan-Meier ten-year survivorship of the prostheses (with survivorship defined as the patient surviving without undergoing revision of the THA).

### Data Extraction and Analysis

We abstracted the following information from each eligible article: the number of patients who had had a primary THA, the calendar years during which the primary THAs were performed, the years during which the cohort was followed, the proportion of patients with osteoarthritis (OA), the definition of revision, the type of fixation method (cemented, uncemented, hybrid or not specified), the number of patients at risk at ten years, and the Kaplan-Meier based probability of revision-free survival at ten years. Additionally, we abstracted the age of the patients and whether the papers reported analyses for certain age brackets. The reports differed in their classification of “younger” patients. “Younger” denotes age less than 60 in one (Norwegian) registry and less than 55 in two (Finnish and Swedish) registries. Two authors abstracted each of the articles included in the review to ensure reliability. Any discrepancies were resolved through discussion with the senior author.

The principal outcome variable for this analysis was the Kaplan-Meier probability of revision-free implant survival at ten years. Some studies provided this parameter and others provided Kaplan-Meier plots from which we estimated the revision-free survival at ten years graphically. Two Swedish studies provided nine-year Kaplan-Meier estimates [12], [13] and one Norwegian study provided eight-year estimates [14]. We transformed these to Kaplan-Meier ten-year estimates by assuming a constant annual risk of revision.

In general, studies reported on revision for any reason. Some of the Swedish [12], [13] and Finnish [15] data report revision for aseptic loosening only. We note these instances in the text, tables and figures.

We used evidence tables and graphical techniques to describe the THA revision risks across national and regional registries and to examine revision risks in relevant subgroups defined by age and fixation technique (cemented vs. uncemented vs. hybrid). In circumstances where Kaplan-Meier ten-year revision-free survival values were given for subgroups defined by fixation status and age category, we derived summary estimates for fixation groups and for age groups by calculating weighted averages of Kaplan-Meier estimates across the relevant subgroups, with weights proportional to the number of patients in each subgroup.

The funding sources for this study had no role in design, analysis or reporting of results.

## Results

### Results of Search

**Figure 1:**
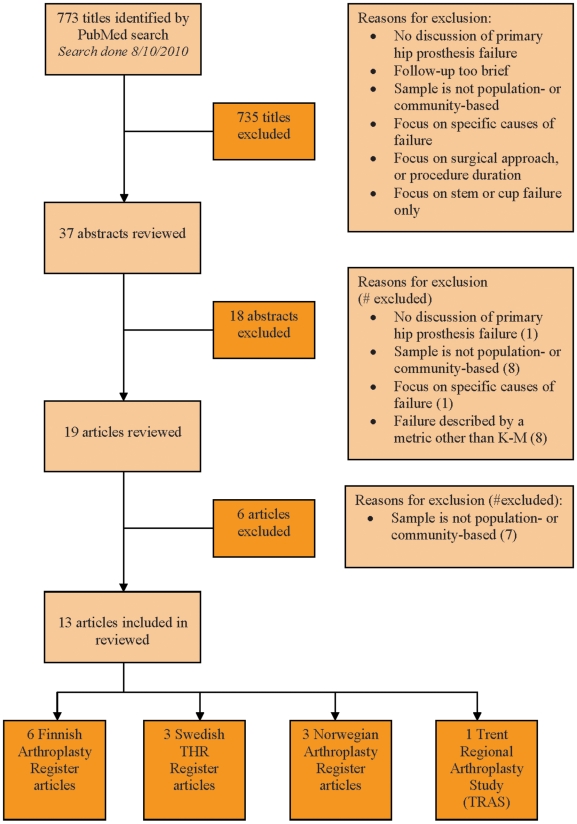
Manuscript search and selection process.

The results of the search for papers on revision of primary THA in national samples are shown in Figure 1. Thirty-seven abstracts were identified. Of these, eighteen were excluded from further consideration because they failed to address hip prosthesis survival (1 abstract), did not rely on population- or community-based samples (8), focused on specific causes of revision (1) or because they provided incidence rates of the primary THA rather than revision rates (8). Nineteen abstracts were eligible for further analysis; these papers were retrieved and reviewed. Of these, six were excluded because the samples were not population- or community-based. Thirteen papers were used as a basis for the current review: six from the Finnish Arthroplasty Register [8], [15], [16], [17], [18], [19], three from the Swedish Total Hip Replacement Register [12], [13], [20], three from the Norwegian Arthroplasty Register [14], [21], [22], and one from the Trent Regional Arthroplasty Study (TRAS) in England [23] (see Table 1). The Swedish, Norwegian, Finnish, and Trent Registers are described in Appendix S1 (see supporting information).

**Table 1:** Overview of papers included in the review.

Paper source (Citation #)	N	Years of operation	Years of follow-up	AR/N	Definition of survival endpoint	Definition of Revision	Country
Allami et al (2006) *Outcome of Charnley total hip replacement (23)*	1,198 (C)	1990	1990–2002	N/A	Revision for any reason	Removal of original components	England
Eskelinen et al (2006) *Uncemented Total Hip Arthroplasty (16)*	1410 (U)	1980–2003	1980–2005*	482/1410(.34)	Revision for any reason	Removal or exchange of any component	Finland
Maleka et al (2008) *Cemented total hip replacement for primary osteoarthritis (8)*	26347(C)	1980–2005	1980–2005*	10645/26347(.40)	Revision for any reason	Removal or exchange of the femoral head, liner or the whole implant	Finland
Makela et al (2008) *THA for primary osteoarthritis in patients fifty-five years or older (17)*	34296 (C)12888 (U)3784 (H)	1980–20041985–20041988–2004	1980–20051985–20041988–2004	10343/34296 (.30)2750/12888 (.21)344/3784 (.09)	Revision for any reason	Exchange or removal of the cup and/or stem or exchange of the liner	Finland
Makela (2010) *Cementless THA for primary osteoarthritis in patients aged 55 years and older (15)*	9,549 (C)10,310 (U)	1980–2005	1980–2005	4447/9549 (.47)2610/10310 (.25)	Revision due to aseptic loosening only	Surgical removal or exchange of the whole or part of the implant	Finland
Ogino et al (2008) *Total Hip Replacement in Patients eighty years of age and older (18)*	5047 (C)399 (U)729 (H)	1980–2004	1980–2004	N/A	Revision for any reason	Removal, exchange, or reimplantation of one, or both, of the prosthetic component	Finland
Puolakka et al (2001) *The Finnish Arthroplasty Register (19)*	4,609 (C)5,519 (U)	1990–1994	1990–2000	N/A	Revision for any reason	Exchange or removal of part of a component, or the whole implant	Finland
Espehaug (2009) *18 years of results with cemented primary hip prostheses in the Norwegian ArthroplastyRegister (14)*	24728 (C)37577 (C)	1987–19971998–2007	1987–20071998–2007	14622/24728 (.59)4298/37577 (0.11)	Revision for any reason	Surgical removal or exchange of the whole or part of the implant	Norway
Furnes et al (2001), *Hip disease and the prognosis of total hip replacements (21)*	37215 (US)11225 (C)	1987–1999	1987–1999	2384/37215 (.06)703/11225 (.06)	Revision for any reason	Removal or exchange of a part of, or the whole implant	Norway
Hallan et al (2007) *Medium- and long-term performance of 11,516 uncemented primary femoral stems from the Norwegian arthroplasty register (22)*	8,444 (U)	1987–2005	1987–2006	N/A	Revision for any reason	Revision of any component (acetabular shell, liner or stem)	Norway
Hailer (2010) *Uncemented and cemented primary THA in the Swedish Hip Arthroplasty Register (20)*	161,413 (C)8,953 (U)	1992–2007	1992–2007	N/A	Revision for any reason	Exchange or removal of any part of the cup or stem, or the entire implant	Sweden
Herberts and Malchau (2000), *Long-term registration has improved the quality of hip replacement (13)*	65,689 (C)2645 (U)	1988–1997	1988–1997	N/A	Revision due to aseptic loosening only	Exchange or removal of one or both components of the prosthesis; Exchange of a liner or head component	Sweden
Malchau et al (2000), *The Swedish THR Register (12)*	2588 (CY)56820(CO)1004 (UY)1083 (HY)	1992–20001992–20001992–20001992–2000	1992–2001*1992–2001*1992–2001*1992–2001*	N/A	Revision due to aseptic loosening only	Exchange or removal of one or both components, or the implant	Sweden

C =  cemented; CO =  cemented old; CY = cemented young; U =  uncemented; UY =  uncemented young; H =  hybrid; HY =  hybrid young; US =  unspecified;

AR/N =  No. at risk at 10 yr/No. of primary operations;

*Estimate because years of follow-up were not given.

### Revision Risk

#### Fixation

**Figure 2:**
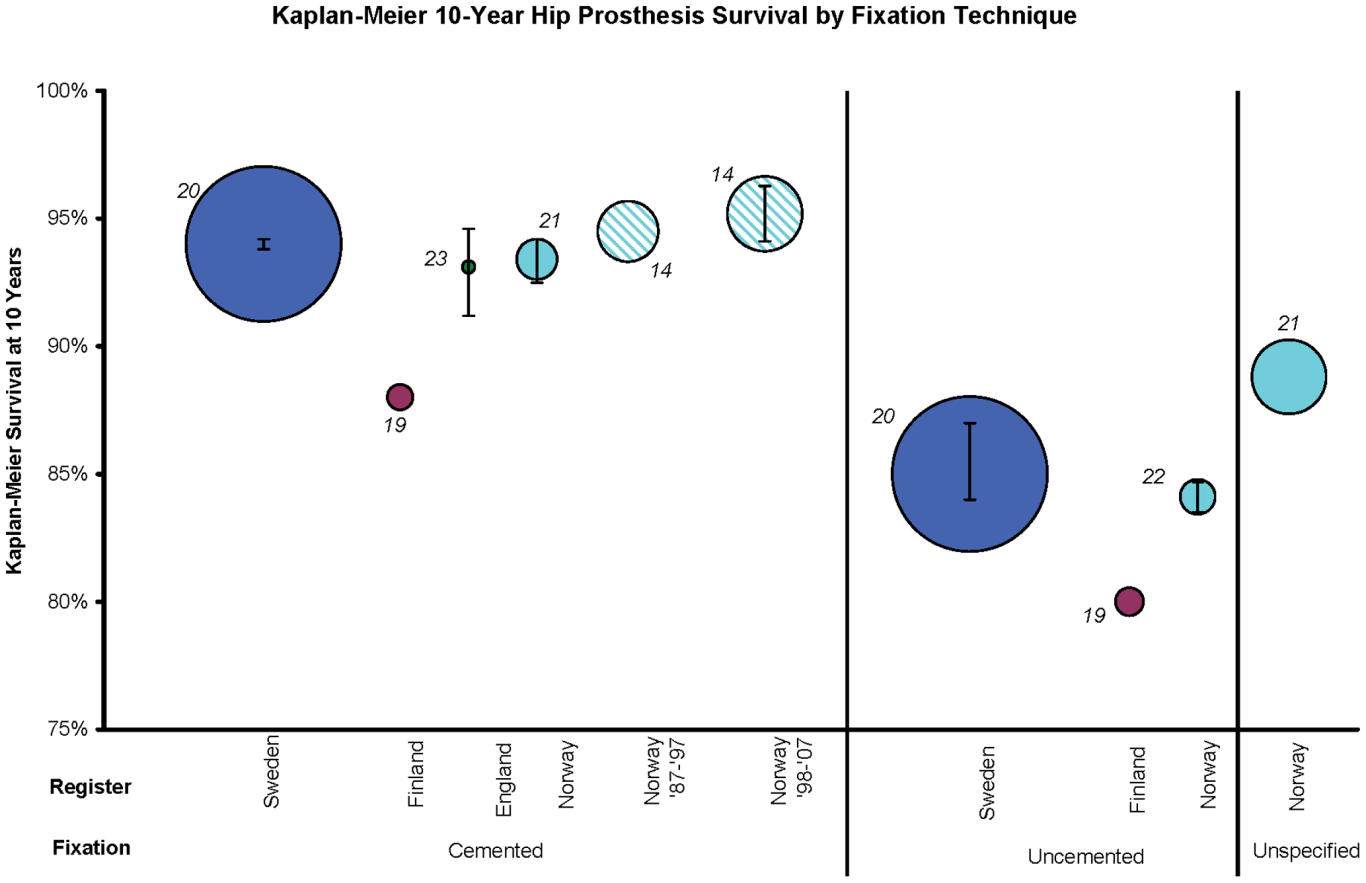
Kaplan-Meier ten-year hip prosthesis survival by fixation technique. Kaplan-Meier ten-year estimates are presented, stratified by national register and fixation technique. Circle area is proportional to the sample size. Error bars represent 95% CIs. All estimates use revision for any reason as the endpoint. Cemented Norway and cemented England are single prosthesis studies, including only Charnley implants. The K-M 10 for uncemented THA in Norway is the weighted average of nine [21], [22] or ten [14] types of implant. All others are inclusive of all prosthesis brands. The Finnish K-M 10 data were estimated from K-M curves. Estimates are inclusive of all patient ages. 31% to 71% of the uncemented Norwegian sample, 87% of the English sample, and 100% of the remaining registers' samples were operated on for osteoarthritis. Each study reference is denoted next to the circle, representing the corresponding manuscript from which the Kaplan-Meier estimates were derived.

Our findings suggest that cemented implants have greater longevity when compared broadly to uncemented implants (Figure 2). Cemented prostheses had ten-year Kaplan-Meier estimates of revision-free survival ranging between 88% (95% CI not provided) in Finland to 95% (95% CI: 94.1, 96.3) in Norway. Uncemented prostheses had higher revision risks across the registers, with Kaplan-Meier ten-year revision-free survival estimates ranging from 80% in Finland (95% CI not provided) to 85% (95% CI: 84, 87) in Sweden. Hybrid prosthesis revision risks were only reported in the Swedish Register, precluding comparison of hybrid prosthesis survival data across countries. In Sweden, hybrid prosthesis survival with revision for aseptic loosening only as the endpoint had a 10-year survival of 92.7% (95% CI: 90.1, 95.4). Osteoarthritis was the only diagnosis included in these estimates of revision risk by fixation, with the exception of the English sample, which was 87% OA, and the uncemented Norwegian sample which ranged from 31 to 71% OA.

#### Age

**Figure 3:**
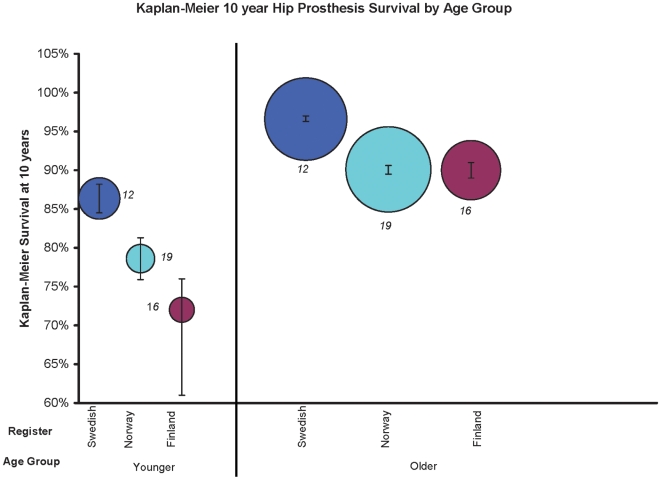
Kaplan-Meier ten-year hip prosthesis survival by age group. Kaplan-Meier ten-year estimates are stratified by register and age group. Circle area is proportional to the sample size. Error bars represent 95% CIs. Estimates are inclusive of all prosthesis types. The endpoint was revision for any reason, except for the Swedish studies, where it was revision due to aseptic loosening only. 75% of the Swedish sample, 78% of the Finnish sample, and 100% of the Norwegian sample were operated on for osteoarthritis. Each study reference is denoted next to the circle, representing the corresponding manuscript from which the Kaplan-Meier estimates were derived.

The Kaplan-Meier ten-year revision-free survival estimates for younger patients ranged from 72% (95% CI: 67, 76) in Finland to 86% (95% CI: 84.5, 88.2) in Sweden (Figure 3). Revision risk was lower in older patients, with Kaplan-Meier ten-year revision-free survival estimates ranging from 90% (95% CI: 89, 91) in Finland to 97% (95% CI: 96.3, 97) in Sweden. The endpoint for these estimates was revision for any reason. These estimates were inclusive of all fixation methods (e.g. cemented, uncemented) and types of prostheses. The Norwegian reports excluded all diagnoses other than OA, while the Swedish sample was 75% OA and the Finnish sample was 78% OA.

#### Fixation and Age

To address the possibility that the main effects for fixation are confounded by age, we examined estimates of prosthesis revision risk in subgroups defined by fixation and age. These analyses are shown in Table 2. In younger and in older patients, cemented implants had lower revision risks than uncemented implants. Among both cemented and uncemented implants, revision risk was lower in older than in younger patients. The endpoint for these estimates was revision for any reason, except in the Swedish data, which report revision due to aseptic loosening only.

**Table 2:** Kaplan-Meier ten-year revision-free survival estimates by Fixation, Age and Register.

Fixation type	Age group	Register	N	% K-M 10	95% CI	Years of operation	Years of follow-up	Age of cohort	% OA
Cemented	Young	Sweden	2,588	88.8	(83.7, 92.7)	1992–2000	1992–2001*	<55	75
	Old	Sweden	56,820	96.2	(95.8, 96.7)	1992–2000	1992–2001*	≥55	75
		Finland	34,296	89.0	(89, 89)	1980–2004	1980–2005	≥55	100
		**Weighted average**	93,704	**93.5**					
Uncemented	Young	Sweden	1,004	94.7	(92.5, 96.9)	1992–2000	1992–2001*	<55	75
		Finland**	1,410	77		1980–2003	1980–2005*	<55	100
		**Weighted average**	2,414	**84.4**					
	Old	Finland∧	7,145	86.0	(85, 88)	1986–2004	1986–2004	≥55	100
		Finland∧	5,743	87.0	(86, 88)	1985–2004	1985–2004	≥55	100
		**Weighted average**	12,888	**86.4**					
Hybrid	Young	Sweden	1,083	92.2	(87.5, 97.1)	1992–2000	1992–2001*	<55	75
	Old	Finland	3,784	88.0	(86, 90)	1988–2004	1988–2004	≥55	100

*Estimate because years of follow-up were not given;

**Comprised from weighted averages of multiple implants;

∧ Note, these two studies from Finland involve different prostheses; the patient samples are independent;

Outcome here is revision due only to aseptic loosening for the Swedish Registry and revision for all indications for the other registries.

## Discussion

Long-term clinical results of THA have been well-documented in the literature. However, the vast majority of studies report findings from individual referral centers. The comprehensive Scandinavian Registers have reported on prosthesis revision rates across entire countries, providing our best estimates of implant revision risk in large, national populations. However, to our knowledge, there has not been a systematic literature review of published articles describing THA survival rates from these large national or regional registries. We have summarized and compared findings from national and regional studies to describe the ten-year prosthesis survival following primary THA. We excluded papers with follow-up shorter than ten years, which prevented us from including data from the US Medicare population and the Danish Register. We did include three papers from Norway and Sweden with reported Kaplan-Meier rates of eight and nine years, respectively, from which we were able to extrapolate ten-year survival data, as described in the Methods section.

The findings suggest that both implant fixation and patient age influence prosthesis revision rates in large population- or community-based samples. Older patients with hip implants had revision-free implant survival rates that exceeded 90% at ten years, while the rates for younger patients ranged from 72 to 86%. Cemented implants had lower revision risk than uncemented implants in both younger and older patients. It is possible, however, that this finding is due to residual confounding by age and activity level, with uncemented implants used in younger, more active individuals.

Referral centers have documented greater than 90% revision-free implant survival at ten years, and greater than 80% revision-free implant survival at 25 years following primary THA [1], [5], [7], [9]. Because of the heterogeneity in patient and hospital factors across large national and regional samples, it is not surprising to find somewhat higher revision rates in national registries than at referral centers. The data provided in the registry studies do not permit adjustment of revision rates for hospital or surgeon characteristics, such as procedure volume.

### Methodological Challenges

Readers should be cautioned when interpreting implant revision data. By ten years postoperatively, the number of patients still at risk for revision THA may be quite small. Thus, revision-free survival estimates may be based on a minority of the entire sample. For example, Furnes et al reported that of 37,215 hips replaced in Norway for OA, inclusive of all ages and prostheses, the Kaplan-Meier ten-year revision-free implant survival estimate was 88.8% with revision for any reason as the endpoint. However, only 2,384 hips were still at risk at ten years, which means the K-M 10 estimate is based upon data from just 6% of the original sample. Patients become censored when they are revised, lost to follow-up or die, and they are seldom followed long enough to contribute ten-year survival data. In this example, the mean follow-up time for the entire cohort was only 4.5 years. Thus, for the latter years of the K-M 10 estimates, only a fraction of the entire cohort was eligible to be analyzed [21]. Only six of the thirteen papers analyzed in this review provided information on the number of patients eligible for Kaplan-Meier analysis at ten years [8], [14], [15], [16], [17], [21]. The proportion of original cohort members eligible for analysis at ten years in these four studies ranged from 6% to 59% (see Table 1).

Implicit in any discussion of revision rates is the definition of failure. The papers we analyzed all used revision as the endpoint. The majority of the papers provided data on revision for any reason, as well as on revision due specifically to aseptic loosening. We focused on revision for any reason, relying on this broader criterion because both patients and policy-makers consider any revision to be important, irrespective of the specific reason for prosthesis failure. However, two reports from the Swedish Register [12], [13] and one report from the Finnish Register [15] only documented revision due to aseptic loosening (see Table 1), and we have indicated in any figure or table legends whether these more narrowly defined revision data have been used. Unless otherwise noted, all reported data compare survival rates in which the endpoint was revision for any reason.

Revision surgery is an unambiguous endpoint for a failed total hip replacement. However, revision rates do not capture implants that have failed clinically but have not been treated surgically. For example, this criterion would miss patients with painful, loose prostheses who do not seek medical attention, choose not to have revision, or are not offered revision because their general health is too unstable. Revision is a blunt measure that gives no information on clinical or radiographic outcome or patient satisfaction. Validation studies on the Swedish Register have indicated that clinical failure rates at ten years, as defined by radiographic loosening in combination with the Harris Hip Score and the Western Ontario and McMaster Osteoarthritis Index (WOMAC), are at least twice as high as the revision rates reported by the Register [4], [13], [24].

As implants and fixation techniques evolve over time, it is important to consider how changes might influence overall rates of revision. In Sweden, for example, modern cementing techniques were introduced in the late 1980s and fully established by the early 1990s. These changes improved the cementing process, and are acknowledged as among the reasons for a ten-year revision-free hip prosthesis survival rate of up to 94% [13]. Modern cementing techniques have only been documented in the Finnish Register since 1996 [8]. We included papers published in 2000 or beyond to reduce the influence of such secular changes.

We limited the review to published studies in order to maintain a consistently high standard of methodological rigor. We acknowledge, however, that several registries in countries outside Scandinavia and England offer valuable unpublished data. For example, although the findings we report from Scandinavian countries document better prosthesis survival in cemented than in uncemented designs, data from the Australian Registry suggest that cemented and uncemented prostheses have similar survival [25]. The Australian Registry was established in 1998 and its website provides revision data on cases operated upon from 1999 to 2008. We did not include the Australian data in our formal analyses because they have not been published in a peer-reviewed journal. The discrepancy between Scandinavian and Australian registries in the performance of cemented vs. uncemented prostheses could reflect differences in control for potential confounders but may also reflect differences across countries in technique, materials, or indications.

The Australian data also show higher survival rates overall than many of the studies we included. Extrapolating from the Australian Registry's eight-year prosthesis survival rate of 95.1% by assuming a constant revision rate yields a ten-year survival rate of 94.1%, which is considerably higher than many of the survival rates reported in the Scandinavian Registries (Table 2). The Australian data reflect a more recent THA prosthesis survival experience, incorporating primary and revision hip replacements performed between 1998 and 2008. Similarly, the New Zealand National Joint Register reports a ten-year survival rate of 93.5% based on procedures performed between 1999 and 2008 [26]. Thus, the improved survival in the Australian and New Zealand data as compared with the Scandinavian experience may point to important secular changes. These observations from the antipodean registries suggest that published registry data may themselves have important limitations that should be appreciated before making broad inferences. The discrepancies between these data sources also urge caution in generalizing the Scandinavian experience.

### Limitations

We acknowledge that these national estimates cannot adjust for differences between implant groups in factors such as activity level and weight, which may affect revision risk. It should also be noted that arthroplasty registers may report OA as the underlying diagnosis in some patients who in fact have mild developmental dysplasia [6], [27]. Patients with dysplasia may have worse outcomes following THA than patients with OA, and this difference might skew the reported survival estimates [6], [21]. Additionally, we recognize the inherent imprecision in estimating K-M 10 revision-free survival from curves when the actual data were not presented in tables [18], [19]. The potential subjectivity of this approach is mitigated in part by having two independent abstractors, with any discrepancies resolved by the senior author.

### Conclusion

These methodological challenges and limitations notwithstanding, we summarized ten-year prosthesis revision rates from international registry-based studies, a task that, to our knowledge, has not been done before. Our findings suggest that older patients who live for ten years following total hip arthroplasty experience a ten-year revision risk of about 10%, while younger patients have a somewhat greater risk of revision. As failure leading to revision is a critically important endpoint of THA from the standpoint of patients, surgeons and policy makers, these data will help anchor discussions of revision risks among these parties. Uncemented implants may be associated with greater revision risks in both age groups, although we cannot exclude confounding by age and activity level. Studies of implant failure are methodologically complex and should be accompanied by discussion of the definition of failure and detailed account of the actual number of subjects at risk at time points of interest.

## Supporting Information

Appendix S1 Description of the Registers surveyed in this study
(0.05 MB DOC)

PRIMSA Flow Diagram S1 (0.06 MB DOC)

PRISMA Checklist S1 (0.08 MB DOC)
