# Supplementary material for: Population-Based Rates of Revision of Primary Total Hip Arthroplasty: A Systematic Review
Source: PLoS One. 2010 Oct 20;5(10):e13520. doi: 10.1371/journal.pone.0013520 (PMC2958142; doi:10.1371/journal.pone.0013520)
Supplement: Appendix S1 — Description of the Registers surveyed in this study (0.05 MB DOC) [file pone.0013520.s001.doc]

**Appendix I: Description of Registers**

Sweden: The Swedish Total Hip Replacement Register began in 1979. Today, information exchange between the reporting units and the Register is conducted through an Internet website. All orthopedic departments in Sweden that perform THA participate voluntarily [1,2]. More than 94% of hip operations performed in Sweden are reported to the Register [3]. The data are checked annually and feedback reports are provided to the clinics from the Register [1]. Information on revisions is derived from medical records and linked to data on the primary arthroplasty by patients’ personal identification number [1].

Norway: Since September 1987, all hospitals voluntarily register primary and revision THA operations, and more than 95% of the procedures are reported to the Norwegian Arthroplasty Register. The revisions are linked to the primary operations in the Register by the patients’ unique national social security number. The surgeon is responsible for completing a one-page form which is mailed to the Register. The Register also receives reports on dates of death and emigration from the Norwegian Population Register. To validate the data, the Register is compared to the National Institute of Hospital Research database. Annual reports are provided to all participating clinics, members of the Norwegian Orthopedic Association, and the health authorities [4].

Finland: The Finnish Arthroplasty Register began in 1980 and is maintained by the National Agency for Medicines. Initially participation was voluntary, but in 1997 hip replacement registration became obligatory. Data from the primary and revision operations are linked using the patients’ unique civic registration number. Additionally, data from the arthroplasty register have been linked to other national registers, including the Finnish Hospital Discharge register and the Population Register Center [4,5]. Currently, over 98% of the operations have been reported to the Register [6].

Trent Regional Arthroplasty Study: Established in 1990, the Trent Regional Arthroplasty Study is the first total joint registry in the UK. Standard registration forms are completed on all procedures by the surgeon and submitted to the study’s center. The data are validated by a clerk who compares the registration database to the hospital records to ensure completeness and accuracy. The region has a population of 4.7 million (1990 census) and nineteen National Health Service hospitals [7]. 97%of the operations in 1990 were captured in the register [8].

1. Herberts P, Malchau H (2000) Long-term registration has improved the quality of hip replacement: a review of the Swedish THR Register comparing 160,000 cases. Acta Orthop Scand 71: 111-121.

2. Malchau H, Herberts P, Eisler T, Garellick G, Soderman P (2002) The Swedish Total Hip Replacement Register. J Bone Joint Surg Am 84-A Suppl 2: 2-20.

3. Soderman P (2000) On the validity of the results from the Swedish National Total Hip Arthroplasty register. Acta Orthop Scand Suppl 71: 1-33.

4. Ogino D, Kawaji H, Konttinen L, Lehto M, Rantanen P, et al. (2008) Total hip replacement in patients eighty years of age and older. J Bone Joint Surg Am 90: 1884-1890.

5. Puolakka TJ, Pajamaki KJ, Halonen PJ, Pulkkinen PO, Paavolainen P, et al. (2001) The Finnish Arthroplasty Register: report of the hip register. Acta Orthop Scand 72: 433-441.

6. Makela K, Eskelinen A, Pulkkinen P, Paavolainen P, Remes V (2008) Cemented total hip replacement for primary osteoarthritis in patients aged 55 years or older: results of the 12 most common cemented implants followed for 25 years in the Finnish Arthroplasty Register. J Bone Joint Surg Br 90: 1562-1569.

7. Allami MK, Fender D, Khaw FM, Sandher DR, Esler C, et al. (2006) Outcome of Charnley total hip replacement across a single health region in England. The results at ten years from a regional arthroplasty register. J Bone Joint Surg Br 88: 1293-1298.

8. Fender D, Harper WM, Gregg PJ (2000) The Trent regional arthroplasty study. Experiences with a hip register. J Bone Joint Surg Br 82: 944-947.
