# Supplementary material for: Population-Based Rates of Revision of Primary Total Hip Arthroplasty: A Systematic Review
Source: PLoS One. 2010 Oct 20;5(10):e13520. doi: 10.1371/journal.pone.0013520 (PMC2958142; doi:10.1371/journal.pone.0013520)
Supplement: PRIMSA Flow Diagram S1 — (0.06 MB DOC) [file pone.0013520.s002.doc]

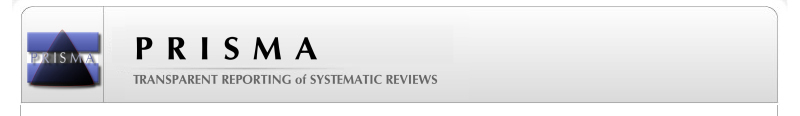
**PRISMA 2009 Flow Diagram**

**Screening**

**Included**

**Eligibility**

**Identification**

Records identified through database searching
(n = 37 )

Additional records identified through other sources
(n = 0)

Records after duplicates removed
(n = 37 )

Records screened
(n = 37 )

Records excluded
(n = 18 )

Full-text articles assessed for eligibility
(n = 19 )

Full-text articles excluded, with reasons
(n = 6 )

Studies included in qualitative synthesis
(n = 13 )

Studies included in quantitative synthesis (meta-analysis)
(n = 10 )
